# Use of Plasma Rich in Growth Factors and ReGeneraTing Agent Matrix for the Treatment of Corneal Diseases

**DOI:** 10.3390/vision5030034

**Published:** 2021-07-02

**Authors:** Ronald M. Sánchez-Ávila, Edmar Uribe-Badillo, Carlos Fernández-Vega González, Francisco Muruzabal, Borja de la Sen-Corcuera, Begoña Baamonde, Luis M. Quirós, Eduardo Anitua, Jesús Merayo-Lloves

**Affiliations:** 1 Fernández-Vega University Institute, Ophthalmology Research Foundation, University of Oviedo, 33071 Oviedo, Spain; edmarub23@gmail.com (E.U.-B.); cfvg@fernandez-vega.com (C.F.-V.G.); bbaamonde@yahoo.es (B.B.); quirosluis@uniovi.es (L.M.Q.); merayo@fio.as (J.M.-L.); 2 Biotechnology Institute (BTI), 01007 Vitoria, Spain; francisco.muruzabal@bti-implant.es (F.M.); bdelasen@bti-health.com (B.d.l.S.-C.); eduardoanitua@eduardoanitua.com (E.A.); 3 University of San Martín de Porres, 15011 Lima, Peru; 4 Biotechnology Institute (BTI), University Institute for Regenerative Medicine and Oral Implantology (UIRMI), 01007 Vitoria, Spain; 5 Department of Functional Biology, University of Oviedo, 33006 Oviedo, Spain

**Keywords:** PRGF, RGTA matrix, ocular surface diseases, PRP, corneal ulcers

## Abstract

This study aimed to investigate the use of Plasma Rich in Growth Factors (PRGF) associated with tissue ReGeneraTing Agent (RGTA) drops for the treatment of noninfectious corneal ulcers. RGTA treatment was applied (one drop every two days); however, if ulcer closure was not achieved, PRGF eye drops treatment was added (four times/day). The time taken to reach the ulcer closure, the Best Corrected Visual Acuity (BCVA), intraocular pressure (IOP), Visual Analog Scale (VAS, in terms of frequency and severity of symptoms), and Ocular Surface Disease Index (OSDI) were evaluated. Seventy-four patients (79 eyes) were included, and the mean age was 56.8 ± 17.3 years. The neurotrophic corneal ulcer was the most frequent disorder (*n* = 27, 34.2%), mainly for herpes virus (*n* = 15, 19.0%). The time of PRGF eye drops treatment associated with the RGTA matrix was 4.2 ± 2.2 (1.5–9.0) months, and the follow-up period was 44.9 ± 31.5 months. The ulcer closure was achieved in 76 eyes (96.2%). BCVA, VAS and OSDI improved from the baseline (*p* < 0.001), and IOP remained unchanged (*p* = 0.665). RGTA and PRGF in noninfectious ulcers were effective and could be a therapeutic alternative for this type of corneal disease.

## 1. Introduction

The ocular surface involves structures such as the cornea, limbus and conjunctiva, which protect ocular integrity against external pathogens under normal conditions [1]. However, non-infectious disorders can generate lesions of varying degrees of severity, from superficial keratitis to ulcers and corneal melting, which can permanently affect visual acuity in many cases [2]. Noninfectious corneal disorders are challenging to diagnose and treat, because they are often associated with systemic diseases, such as autoimmune disorders such as rheumatoid arthritis or metabolic conditions such as diabetes mellitus. In many cases, the ocular environment is predisposed to developing ocular diseases such as atopic conjunctivitis and severe dry-eye. These disorders lead to increased metalloproteinases and pro-inflammatory molecules, which promotes an exacerbation of the corneal tissue destruction [3].

Ocular lubricants, steroidal anti-inflammatory drugs, and immunomodulators are the most common therapies used to treat ocular surface diseases to enhance and restore ocular surface homeostasis. However, the long-term use of corticosteroids can cause undesirable adverse effects, such as super-infections, intraocular pressure (IOP) increase, and cataracts, among others. In this sense, it is essential to avoid the use of corticosteroids in a chronic manner [4]. The use of calcineurin-inhibiting agents, such as topical cyclosporine, is an option to treat ocular surface and corneal diseases, where immunomodulation is affected, without a risk of increased ocular pressure. However, its long-term use remains doubtful in cases where there are recurrent infections, such as herpes virus infection [5].

One attractive alternative to treating ocular surface diseases is blood derivative products, obtained with closed manufacturing protocols [6]. The use of platelet activators allows for the release of a wide variety of growth factors and molecules involved in ocular tissue regeneration. Plasma rich in growth factors (PRGF) is an improved type of platelet-rich plasma (PRP) that was widely used in the treatment of several pathologies in the ophthalmology field [6]. PRGF has some interesting biological properties, such as lubricating, regenerative, immunomodulatory, and bacteriostatic properties; besides this, the safety of PRGF treatment has been broadly demonstrated in several clinical and pre-clinical studies [7,8,9].

The role of the ReGeneraTing Agent matrix (RGTA) eye drops (Cacicol20, OTR3, Paris, France) on the ocular surface and cornea is also known. These eye drops are composed of a polysaccharide that regulates the extracellular matrix-like heparan sulfate. RGTA has shown successful results in the treatment of several corneal surface disorders, including noninfectious ulcers [2,10]. This retrospective study describes, for the first time, the efficacy and safety of both regenerative therapies (PRGF and RGTA), used simultaneously, for the treatment of ocular surface disorders.

## 2. Materials and Methods

### 2.1. Study Desing

A retrospective design study was performed, including patients diagnosed with corneal and ocular surface diseases. The study evaluated the medical records of the patients of the Fernández-Vega University Institute (Oviedo, Spain) between October 2010 and July 2019. The principles of the Declaration of Helsinki were followed to perform this study. All patients included in the present study signed an informed consent for the use of PRGF therapy, and the cases were identified in the electronic patient registry.

Patients included in this study needed to fulfil the criteria for ocular surface disease without infectious activity and with an associated corneal ulcer. The diagnoses of the ocular surface disorders included neurotrophic corneal ulcer (NCU, stage 3 by Mackie classification) from different etiologies, severe dry-eye disease (DED) (Schirmer test I values less than 5 mm, tear film breakup time (TBUT) film values less than 5 s, subjective symptoms of severe dry-eye, and associated corneal ulcer), persistent epithelial defects (PED), exposure keratopathy, corneal ulcer from chemical burns, and epithelial dystrophies, among others. All enrolled patients had been diagnosed with these diseases for at least six months, with no improvement after the use of conventional treatments such as artificial tears, topical/oral corticosteroids, topical/oral antibiotics, topical/oral antivirals, therapeutic contact lenses, punctual occlusion plugs, autologous serum (AS), topical cyclosporine, or amniotic membrane transplant. Patients with hereditary, metabolic, or degenerative retinal diseases were excluded. Patients with active conjunctival, corneal or intraocular infection of bacterial, viral, fungal or protozoan origin were excluded in this study.

### 2.2. Treatment

Patients were initially treated with RGTA eye drops therapy alone (Cacicol20, OTR3, Paris, France) once every two days for three weeks. However, if an improvement in DED symptoms and closure of the corneal ulcer was not achieved, PRGF eye drops treatment was added topically four times daily for six weeks (1 cycle = 6 weeks). Additional cycles of PRGF eye drops were administered according to the evolution in the closure of the corneal ulcer. The RGTA matrix was discontinued when complete corneal ulcer closure was achieved; however, PRGF eye drops therapy was maintained for at least six weeks more to achieve stability in DED symptoms. Patients received other topical treatments (artificial tears or corticosteroids) when they were considered necessary by the physician. Any adverse events or complications that appeared during the study were recorded to evaluate the safety of these treatments.

### 2.3. PRGF Preparation

PRGF eye drops were applied according to the manufacturer´s protocol (Biotechnology Institute (BTI), S.L., Miñano, Álava, Spain) [9]. Briefly, peripheral blood was drawn from patients and collected in 9 ml tubes, then it was centrifuged, and the whole PRGF column was collected after centrifugation, avoiding collecting the layer containing the leukocytes. Then, the PRGF fraction was activated with 10% calcium chloride; the obtained supernatant was filtered and distributed into individual containers and stored at −20 °C until use. Patients were instructed to store the PRGF eye drop dispensers at −20 °C for a maximum of 6 months, and each dispenser had to be used for three consecutive days. The PRGF eye drops were applied topically in the conjunctival sac four times daily for six weeks (one cycle) in the affected eye.

### 2.4. Follow-Up Period

The patients were initially followed weekly for the first month, then every two weeks until the corneal wound was closed. Patients were followed until the closure of the corneal ulcer/defect and the control of DED symptoms were achieved. Data from the clinical records, photographs and questionnaires were collected during the follow-up period.

### 2.5. Outcomes Measures

Demographic data (sex, age, affected eye) and type of corneal disease were collected. A complete eye examination was carried out before the enrollment and during the follow-up with a slit lamp (anterior segment and cornea, fundus), IOP measured by Perkins tonometer, DED symptoms, fluorescein dye test and the characteristics of the corneal wound.

The main outcome measure was the time to achieve corneal wound closure, defined as the number of PRGF eye drops cycles associated with the RGTA matrix until wound closure was achieved. The corneal wound evaluation was performed by instilling sodium fluorescein (2.5%) in the conjunctival sac, and the capture of digital corneal photography under a slit lamp (Emedio^®^, Seoul, Korea). Finally, the corneal defect/ulcer diameter (in millimeters) was measured using the Image J software (ImageJ; http://imagej.nih.gov/ij/; Accessed on 25 July 2019; provided in the public domain by the National Institutes of Health, Bethesda, MD, USA). Corneal staining area was also evaluated with fluorescein staining (0 to 3; 0 = no dotted staining, 1 = staining less than 1/3 of the area, 2 = between 1/3 and 2/3 of the area, 3 = more than 2/3 of the area), and corneal staining density (scale from 0 to 3; 0 = no dotted staining, 1 = sparse density, 2 = moderate density, 3 = high density, with overlapping corneal lesions). Patients were followed for at least six months after corneal wound closure to assess the stability in ocular surface symptoms.

Several secondary outcome variables were studied to evaluate the ocular surface symptoms, including the Visual Analog Scale ((VAS, Frequency and severity of symptoms), estimating a range between 0 to 100, where 0 = no discomfort and 100 = maximal discomfort (dryness, burning/stinging, photophobia, foreign body sensation, blurred vision, itching and pain)); Ocular Surface Disease Index (OSDI) Spanish version that includes 12 questions [10]; and DED grade ranging from 1 to 4 according to DEWS II (Dry Eye WorkShop) recommendations [11]. The Best Corrected Visual Acuity (BCVA) was determined using the Snellen optotype (conversion to logMAR scale-logarithm of the minimum angle of resolution) and IOP in mmHg.

### 2.6. Statistical Analysis

Descriptive statistics were performed using absolute and relative frequency distributions for qualitative variables and mean values and standard deviations. The normal distribution of each variable sample was analyzed using the Kolmogorov–Smirnov and Shapiro–Wilk. The difference before and after treatment was analyzed using the Wilcoxon non-parametric statistical test for non-parametric variables and the Student’s *t*-test for parametric variables. The statistical level of significance was established at *p* < 0.05. Statistical software SPSS v. 19.0 for Windows (SPSS Inc., Chicago, IL, USA) was used for all statistical analyses.

## 3. Results

The present study included seventy-nine eyes from 74 patients affected by different ocular surface and corneal diseases with associated ulcers. The origin of the most frequent corneal ulcer was the NCU (*n* = 27, 34.2%); the second most frequent corneal ulcer was due to severe DED (*n* = 22, 27.8%), followed by exposure keratopathy (*n* = 12, 15.2%) (Table 1). In patients with autoimmune diseases (*n* = 17, 23.0%), oral immunomodulatory treatment was maintained throughout the study exactly as it was being administered before the start of the study.

The mean treatment time with PRGF eye drops associated the RGTA matrix was 4.2 ± 2.2 (1.5–9.0) months to achieve closure of the corneal ulcer. The average of PRGF cycles was 2.6 ± 1.4 (1.0–6.0). The NCU patients (34.2% of the total cases) received a mean number of PRGF cycles of 2.6 ± 1.2 (1.0–6.0) to achieve closure of the corneal ulcer; 1.9 ± 0.8 (1.0–4.0) PRGF cycles were required for severe dry-eye corneal ulcers, 3.3 ± 1.4 (1.0–6.0) for exposure keratopathy, and 2.0 cycles of PRGF eye drops were needed for those caused by chemical burns (Table 2).

**Figure 1:**
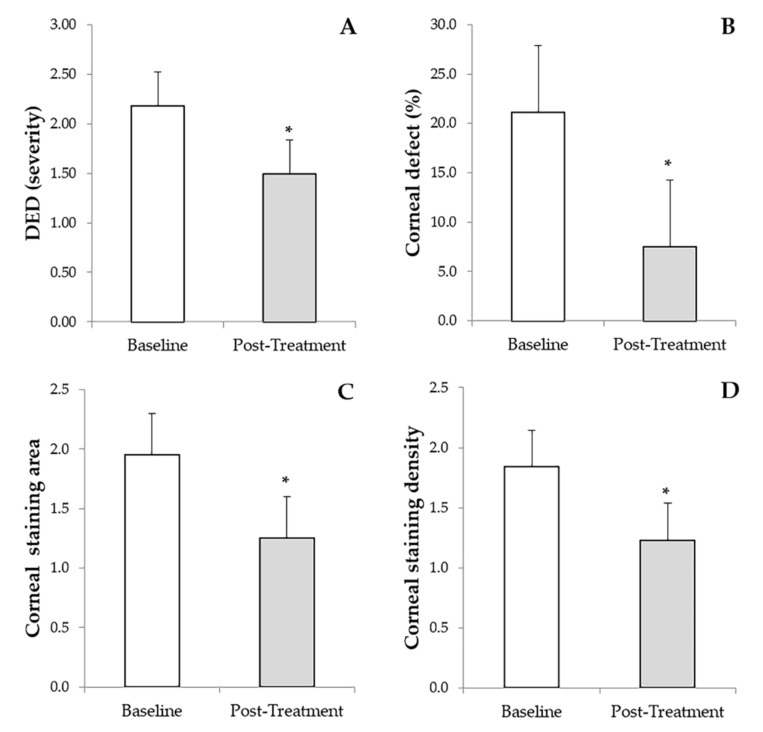
Results of the primary outcome measures before and after treatment with PRGF eye drops and RGTA. (**A**): Dry eye disease (DED). (**B**): Percentage of the corneal defect. (**C**): Corneal staining area. (**D**): Corneal staining density. * Statistically significant differences (*p* < 0.05) before versus after treatment with PRGF and RGTA.

The grade of DED was significantly reduced (*p* < 0.01) from an initial value of 2.18 ± 0.84 (1.0–4.0) to a final post-treatment value of 1.49 ± 0.7 (0.0–3.0) (Figure 1A). PRGF eye drops reduced significantly (*p* < 0.01) the percentage area of the corneal defect from a baseline value of 21.1 ± 8.7 (10.0–40.0) to a final value of 7.5 ±5.9 (0.0–20.0) (Figure 1B). A significant reduction (*p* < 0.001) was observed in the corneal staining area from the baseline value of 1.84 ± 0.78 (1.0–3.0) to a final post-treatment value of 1.25 ± 0.49 (0.0–2.0) (Figure 1C). In addition, corneal staining density improved significantly (*p* < 0.001) from the initial value of 1.84 ± 0.78 (1.0–3.0) to a final follow-up time value of 1.23 ± 0.61 (0.0–2.0) (Figure 1D). The overall mean follow-up of patients was 44.9 ± 31.5 months. Ulcer closure was achieved in 76 eyes (96.2%), and in only three cases did ulcer closure not occur, but there was a closure of 53%, 55% and 64% of the original ulcer, respectively.

**Figure 2:**
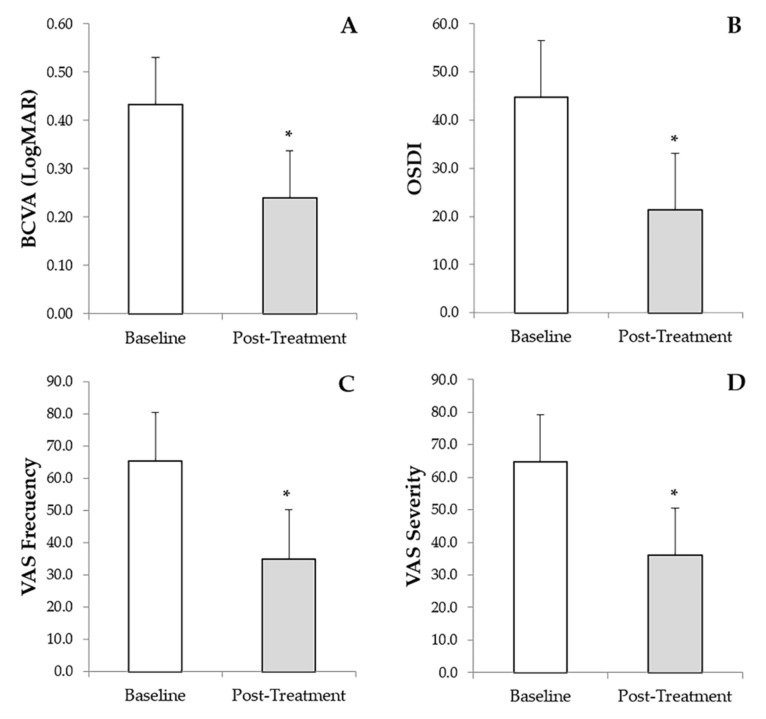
Results of the secondary outcome measured before and after treatment with PRGF eye drops and RGTA. (**A**): Best-corrected visual acuity (BCVA). (**B**): Ocular surface disease index (OSDI). (**C**): Visual analog scale (VAS) frequency, and (**D**): VAS severity. * Statistically significant differences (*p* < 0.05) before versus after treatment with PRGF and RGTA.

The BCVA had an overall improvement of 62.3%. The greatest BCVA improvements were for patients with NCU, exposure keratopathy, and Cogan’s epithelial dystrophy, with 70.7%, 83.4%, and 79.3%, respectively. Additionally, patients with severe DED, post-infection corneal ulcer, Salzmann’s nodular degeneration and Filamentary keratitis showed a median BCVA improvement of 54.6%, 66.7%, 57.6% and 56.3%, respectively. The ocular disorders with less improvement in BCVA values were corneal ulcer from chemical burns with 40.3%, followed by corneal ulcers from PED with 33.3%, chronic cicatricial conjunctivitis and peripheral ulcerative keratitis with 28.8% and 28.3%, respectively. Overall, BCVA improved significantly (*p* < 0.001) from an initial value of 0.434 ± 0.642 (0.000–3.000) to a final post-treatment value of 0.240 ± 0.442 (0.000–2.800) (Figure 2A). Additionally, a significant reduction (*p* < 0.001) was observed in the OSDI scale from a baseline value of 44.8 ± 19.9 (12.5–95.8) to a final follow-up time value of 21.4 ± 13.9 (0.0–47.9) (Figure 2B). Furthermore, regarding the frequency and severity of DED symptoms measured by VAS, a significant decrease (*p* < 0.001) was observed in both variables evaluated, showing a reduction from a baseline value of 65.4 ± 21.1 (20.0–100.0) to a final post-treatment value of 35.0 ± 14.5 (0.0–50.0) in the case of VAS frequency (Figure 2C), and from an initial value of 64.8 ± 19.3 (20.0–100.0) to a final post-treatment value of 36.1 ± 13.6 (0.0–50.0) in the case of VAS severity (Figure 2D). Finally, IOP values did not change from baseline (13.5 ± 3.9 mmHg) to the final follow-up period (13.5 ± 1.7). No adverse events were recorded regarding the use of PRGF eye drops, nor with the use of the RGTA matrix. No additional topical treatment with corticosteroids, antibiotics, or artificial tears was required throughout the study follow-up.

**Figure 3:**
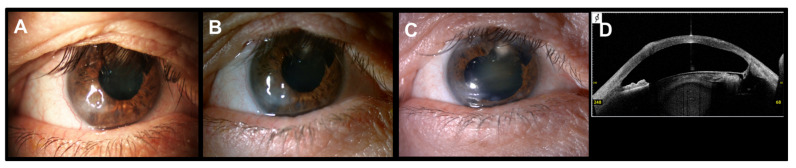
Corneal ulcer of a patient with Salzmann’s nodular degeneration treated with PRGF eye drops and RGTA. (**A**): Baseline situation. (**B**): Corneal image obtained after one month of follow-up, the corneal ulcer is under closure process. (**C**): Complete corneal ulcer closure after three cycles of PRGF eye drops. (**D**): OCT-SA image, restoration of corneal integrity maintained along the follow-up period (80.5 months).

**Figure 4:**
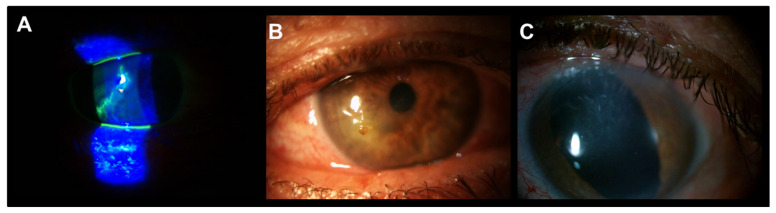
Patient with meta-herpetic corneal ulcer treated with PRGF and RGTA. (**A**): Baseline image. (**B**): Acute post-infection neurotrophic corneal ulcer. (**C**): Complete corneal ulcer closure after two cycles of PRGF eye drops. The corneal closure was stable throughout the follow-up (38.3 months).

The patient with Salzmann’s nodular degeneration required three cycles of PRGF eye drops to achieve complete ulcer closure (Figure 3). Figure 4 shows one of the cases of NCU due to herpes virus in which two cycles of PRGF eye drops were required to achieve ulcer closure after a total follow-up of 38.3 months.

## 4. Discussion

Noninfectious corneal ulcers are laborious to diagnose, and, in some cases, conventional treatment (lubricants, topical immunomodulators, ointments) is insufficient. This is due to the delay in identifying a noninfectious-type disease and, in many cases, due to the exacerbation of clinical findings when using first-line empirical antibiotic treatment [12].

In the present study, the most frequent cause of noninfectious ulcers was neurotrophic keratitis, which occurs due to a corneal sensory alteration, thus inducing damage to the corneal surface and is associated with a reduction in the production and quality of the tear [12]. In many cases, these patients have a history of ocular trauma, including surgery, or sequelae of infections like the herpes virus. The usual treatment for this type of disorder is intended to achieve the ulcer’s re-epithelialization once the previous or coexisting infectious pathology has been ruled out. In this case, several treatments such as preservative-free artificial tears, therapeutic contact lenses, eye patches with epithelizing ointments, anti-collagenase therapy (Tetracyclines, N-acetylcysteine, Medroxyprogesterone), or amniotic membrane transplant, among others, are used [13]. However, many of these therapies alone or in combination, cannot completely restore the cornea and maintain tear film homeostasis.

Severe DED was the second cause of corneal ulcers in this study. Most of these patients had been treated with chronic therapies like artificial tears, immunomodulators, or epithelializing ointments for a long time without achieving clinical improvement, which shows how difficult it is to treat these patients [7].

The use of blood derivatives in treating noninfectious alterations of the ocular surface and cornea is not new [14]. The AS was used for the treatment of DED refractory, and since then, there have been many studies and reviews about the possible use of AS for the treatment of ocular surface pathologies, with different results. In 2017, Pan et al. concluded that the use of AS in patients with DED improved symptoms in the first 15 days of treatment and, after that, no differences were found compared to the use of artificial tears [14]. Non-standardization in the manufacture of AS (variable protocols), variability in clotting time, centrifugation speed, or different dilution percentages (from 15 to 40%) are the main problems in the manufacture of AS, which leads to variable results in the treatment of ocular surface disorders [14].

PRGF is an alternative for the treatment of patients with corneal disorders with a noninfectious cause. It is known that PRGF contains a broad spectrum of molecules and growth factors that are involved in cell differentiation, proliferation, and migration such as epithelial growth factor (EGF), platelet-derived growth factor (PDGF), fibronectin, vitamin A [15], some trophic factors [8], anti-inflammatory agents [7] and bacteriostatic and bactericidal molecules [16]. The production of PRGF is performed with a closed and standardized manufacturing protocol, which provides stability to its product for up to six months after its preparation [17,18]. Nonetheless, unlike other blood products such as AS, PRGF does not contain leukocytes, which could induce undesirable effects on the treatment of several ocular pathologies due to the inflammatory cytokines released by them, especially in autoimmune diseases patients [9,19].

On the other hand, the effectiveness of RGTA was demonstrated in the closure of NCU [2,20], and for the treatment of accidental injuries (cornea burns) [21]. The RGTA matrix was also shown to be useful in the closure of corneal ulcers in an animal model of photorefractive keratectomy (PRK) surgery [22].

In this clinical study, the regenerative additive effect of the RGTA matrix and PRGF eye drops are studied in patients who do not respond adequately to previous treatment with the RGTA matrix alone. The combined use of both therapies reached a high degree of success, thus achieving a total closure of the corneal ulcer in 96.2% of the evaluated eyes. In a previous study, the use of PRGF alone demonstrated the closure of corneal ulcers in 97% of cases in patients with NCU who were non-responders to the previous treatment [23]. This study raises the possibility of using the RGTA matrix, which hypothetically would serve as a scaffold that allows the absorption and subsequent sustained release of the growth factors provided by PRGF, although more studies are required to confirm these clinical findings.

In this study, a statistically significant decrease (*p* < 0.01) was observed in the different variables measured, such as the percentage of the corneal defect, corneal staining area, corneal staining density, VAS frequency, VAS severity, degree of DED and OSDI. Similar results were found in previous publications where PRGF alone improved the values of discomfort in patients with ocular surface disorders [1,20]. These successful results could be due in part to the characteristics of PRGF, such as its analgesic or anti-inflammatory effect, which has been widely demonstrated in previous studies [24]. In the present study, patients had a clinical follow-up period of more than one year, and no adverse events related to the applied therapy were recorded; hence it suggests that the combined therapy of RGTA matrix and PRGF eye drops is effective and safe for the treatment of noninfectious ocular surface disorders.

There are other alternatives for the treatment of noninfectious corneal defects, including topical insulin, which has been used since the 1940s [25], and other recent therapies for the treatment of NCU [26]. The use of Substance P associated with Insulin-like growth factor 1 (IGF-1) has been shown to have a synergistic effect on the epithelization process of corneal ulcers, especially those of neurotrophic origin [27]. One of the most recent and promising therapies is the use of recombinant human nerve growth factor (rh)NGF (Cenegermin), which has demonstrated its efficacy in the closure of NCU, obtaining the approval by the European Medicines Agency (EMA) for ophthalmological use; however, its widespread use has not yet become popular [28].

As far as we know, this is the first clinical study combining the treatment of the RGTA and PRGF matrix to treat corneal ulcers. There are some limitations in this study, including the fact that it is a retrospective and uncontrolled study; however, it opens the way for future multicenter studies to confirm our results.

## 5. Conclusions

The use of RGTA matrix and PRGF eye drops is effective and safe for treating noninfectious corneal ulcers. Furthermore, PRGF treatment could be associated with other therapies to improve the closure of corneal ulcers of different ocular surface pathologies.

## Figures and Tables

**Table 1:** Baseline characteristics of patients treated with PRGF and RGTA matrix.

Patients/Eyes	74/79
**Affected eye**	
RE, n (%)	36 (45.6)
LE, n (%)	43 (54.4)
**Gender**	
Female, n (%)	46 (62.2)
Male, n (%)	28 (37.8)
**Years**	
Overall, mean ± SD (range)	56.8 ± 17.3 (25.9–87.8)
Female, mean ± SD (range)	55.7 ± 17.7 (25.9–86.6)
Male, mean ± SD (range)	58.6 ± 16.7 (30.2–87.8)
**Corneal disorders**	
Neurotrophic corneal ulcer, n (%)	27 (34.2)
*Herpetic*, *n* (*%*)	*15* (*19.0*)
*Post-LASIK*, *n* (*%*)	*4* (*5.1*)
*Diabetes mellitus*, *n* (*%*)	*3* (*3.8*)
*Traumatic*, *n* (*%*)	*1* (*1.3*)
*Post-PRK*, *n* (*%*)	*1* (*1.3*)
*Anti-glaucoma eye-drops*, *n* (*%*)	*1* (*1.3*)
*GVHD*, *n* (*%*)	*1* (*1.3*)
*Post-phaco*, *n* (*%*)	*1* (*1.3*)
Severe DED, n (%)	22 (27.8)
Exposure keratopathy (ulcer), n (%)	12 (15.2)
Causticization corneal ulcers, n (%)	5 (6.3)
Persistent epithelial defect, n (%)	3 (3.8)
Post-infection corneal ulcer, n (%) *	3 (3.8)
Chronic cicatricial conjunctivitis, n (%) †	2 (2.5)
Peripheral ulcerative keratitis, n (%)	2 (2.5)
Cogan’s epithelial dystrophy, n (%)	1 (1.3)
Salzmann’s nodular degeneration, n (%)	1 (1.3)
Filamentous keratopathy, n (%)	1 (1.3)

PRGF: Plasma Rich in Growth Factors, RGTA: ReGeneraTing Agent matrix, RE: Right, LE: Left eye, SD: Standard deviation, GVHD: Graft Versus Host Disease, DED: Dry-eye disease. * One eye with persistent ulcer after the resolution of the corneal abscess, two eyes with persistent ulcers after adenovirus infection. † Two eyes (1 patient) with mucous membrane pemphigoid with eye affection.

**Table 2:** Time and number of cycles of PRGF eye drops treatment and follow-up period.

Corneal Disorder	Number of PRGF Cycles (Mean ± SD (Range))	PRGF Treatment in Months (Mean ± SD (Range))	Clinic Follow-Up in Months (Mean ± SD (Range))
Neurotrophic corneal ulcer	2.6 ± 1.2 (1.0–6.0)	3.9 ± 1.8 (1.5–9.0)	43.4 ± 30.5 (0.3–113.4)
Severe dry eye disease	1.9 ± 0.8 (1.0–4.0)	2.9 ± 1.3 (1.5–6.0)	46.0 ± 34.5 (0.9–118.8)
Exposure keratopathy (ulcer)	3.3 ± 1.4 (1.0–6.0)	5.6 ± 2.4 (1.5–9.0)	54.8 ± 33.8 (1.8–120.8)
Corneal ulcer from chemical burns	2.0 ± 0.0 (2.0–2.0)	3.0 ± 0.0 (3.0–3.0)	38.2 ± 33.3 (2.3–68.7)
Persistent epithelial defect	2.7 ± 0.6 (2.0–3.0)	4.0 ± 0.9 (3.0–4.5)	42.8 ± 28.0 (10.5 –61.1)
Post–infection corneal ulcer	2.0 ± 0.0 (2.0–2.0)	4.5 ± 2.6 (3.0–7.5)	36.1 ± 19.6 (14.5–52.7)
Chronic cicatricial conjunctivitis	6.0 ± 0.0 (6.0–6.0)	9.0 ± 0.0 (9.0–9.0)	35.9 ± 42.1 (6.1–65.6)
Peripheral ulcerative keratitis	4.0 ± 2.8 (2.0–6.0)	6.0 ± 4.2 (3.0–9.0)	33.5 ± 45.5 (1.3–65.7)
Cogan’s epithelial dystrophy	4.0	6.0	6.3
Salzmann’s nodular degeneration	3.0	4.5	80.5
Filamentous keratopathy	6.0	9.0	49.0
Total	2.6 ± 1.4 (1.0–6.0)	4.2 ± 2.2 (1.5–9.0)	44.9 ± 31.5 (0.3–120.8)

PRGF: Plasma Rich in Growth Factors, SD: Standard deviation.

## Data Availability

All data were fully anonymized and are available upon request.
